# Level of underreporting including underdiagnosis before the first peak of COVID-19 in various countries: Preliminary retrospective results based on wavelets and deterministic modeling

**DOI:** 10.1017/ice.2020.116

**Published:** 2020-04-09

**Authors:** Steven G. Krantz, Arni S.R. Srinivasa Rao

**Affiliations:** 1 Department of Mathematics, Washington University, St Louis, Missouri; 2 Division of Health Economics and Modeling, Department of Population Health Sciences, Medical College of Georgia, Augusta University, Augusta, Georgia; 3 Laboratory for Theory and Mathematical Modeling, Department of Medicine - Division of Infectious Diseases, Medical College of Georgia, Augusta, Georgia; 4 Department of Mathematics, Augusta University, Augusta, Georgia

We estimated the underreporting of the novel coronavirus or COVID-19 as of March 9, 2020, in various countries until the first peak occurred in each country that had reported ≥500 cases of COVID-19 as of March 9, 2020. Our retrospective model-based estimations of underreporting (including those due to underdiagnosis) will be helpful in assessing pandemic preparedness. The ratio of reported COVID-19 cases to model-based predictions of COVID-19 for 8 major countries that had reported ≥500 cases up to March 9, 2020, are provided (Table 1, column l). COVID-19 reporting in France, Germany, Italy, and South Korea was comparatively much better than in other countries. For the United States, the data as of March 9, 2020, were not sufficient to provide a robust estimate.

**Table 1.** COVID-19 Cases, Demographics, Daily Cases, Growth Rates, and Estimated Underreporting up to March 9, 2020

Country by No. of Confirmed Cases	Total COVID-19 Cases	Total Deaths	Population Density (2020), km^–^^2^	Urban Population, (2020), %	Date Range of Daily New Cases up to the First Peak	Range of Daily New Cases up to the First Peak	Population Aged 0–14 y (2018), %	Population Aged 15–65 y (2018), %	Population Aged ≥65 y (2018), %	Model-Based Underreported & Underdiagnosed up to March 9, 2020	No. of People Reported to the No. Infected
China	80,761	3,136	153	61	Jan 22–Feb 4	259–3,884	17.9	71.2	10.9	12.03–89.2 million	1 in 149 to 1 in 1,104
Italy	10,149	631	206	69	Feb 22–Mar 9	58–1,797	13.3	64.0	22.7	30,223	1 in 4 reported
Iran	8,042	291	52	76	Feb 21–Mar 6	13–1,234	24.5	69.3	6.2	266,213	1 in 34 reported
South Korea	7,513	58	527	82	Feb 23–Mar 3	27–851	13.0	72.6	14.4	18,809	1 in 4 reported
France	1,784	33	119	82	Feb 27–Mar 7	20–296	18.0	62.0	20.0	7,931	1 in 5 reported
Spain	1,690	35	94	80	Feb 27–Mar 9	12–557	14.7	66.0	19.3	87,405	1 in 53 reported
Germany	1,458	2	240	76	Feb 27–Mar 5	22–283	13.6	65.0	21.4	2,277	1 in 3 reported
United States	874	28	36	83	Mar 2–Mar 12	25–1,652	18.7	65.5	15.8	1.21 million (insufficient data)	1 in 406 reported (insufficient data)

According to Situational Report 49, released by the World Health Organization (WHO) on March 9, 2020,^1^ there had been 109,000 cases of COVID-19 and 3,800 related deaths worldwide. Most of these cases (~80,700) were from China and 8 other countries: Italy, South Korea, Iran, France, Germany, Spain, the United States, and Japan. All of these countries have reported ≥500 confirmed cases of COVID-19.^1,2^ However, identification of possible cases of COVID-19 is arguably more important in controlling high traffic to hospitals and emergency departments.^3^ Earlier models on COVID-19 did reflect the importance of data collection.^4^

Actual pandemic preparedness depends on true cases in the population, whether or not they are identified. Preventing transmission to the susceptible from these true cases depends on how well we can assess underreported and underdiagnosed situations promptly. A retrospective analysis of the data will be useful for the next epidemic but not for the current epidemic. Hence, we are proposing to use our methods, which we have been developing in recent years, to provide model-based estimates of underreporting for COVID-19 within a few weeks.

New methods using harmonic analysis and wavelets that we are developing—some of them recently accepted—will be of timely use.^5^ We propose a model-based evaluation of underreporting of coronavirus (COVID-19) in various countries using the methods we recently developed using harmonic analysis,^5^ that is, to develop full epidemic data from partial data (using a wavelet approach). However, the current article is a preliminary analysis and modeling was done using the data available as of March 9, 2020. These data do not represent the pandemic in its entire scale; such data will need to be reevaluated when the pandemic is completely controlled. However, our predictions for underreporting as of March 9 in a couple of European countries were close to the reported number of COVID-19 cases as more cases surfaced from March 9 to March 16, 2020. Wavelets of reported cases and adjusted estimates with the underreported cases are shown in Figure 1. We also anticipate using other techniques^5–9^ to further understand the reporting once more data become available.

**Figure 1.**
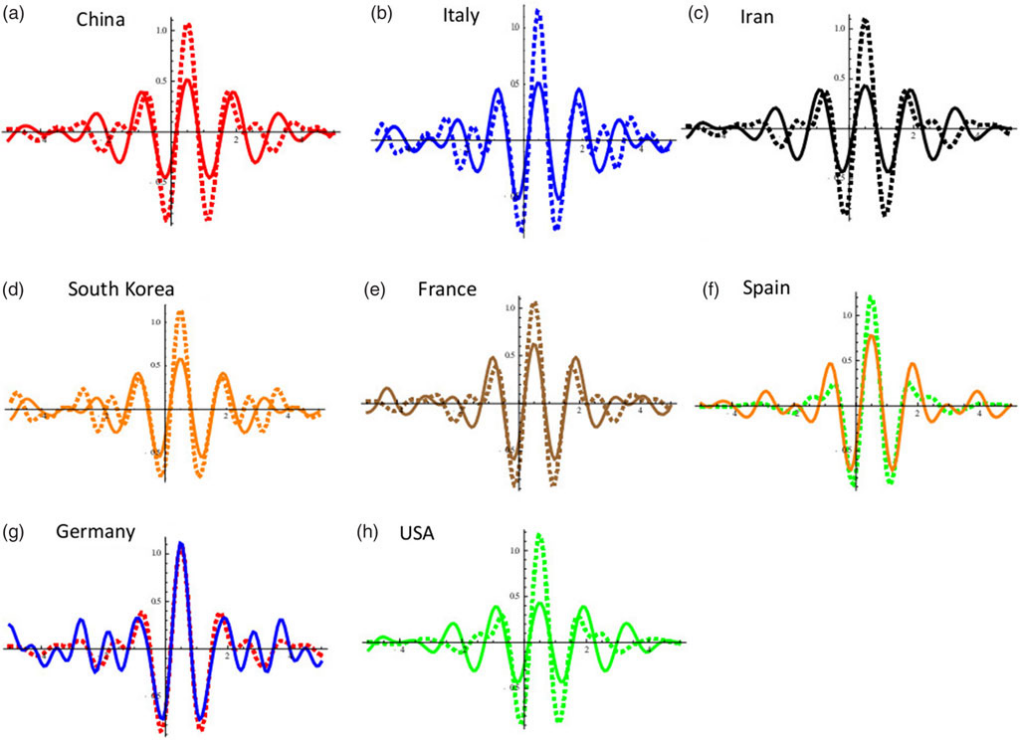
Meyer wavelets for various countries for reported (dashed lines) and adjusted data after adjusting for under-reporting listed in the Table 1.

## Data, Methods, and Models

We collected COVID-19 and population data for each country from the World Health Organization (WHO),^1^ Worldometer,^2^ and World Bank^10^ sources. We used population densities, proportion of the population living in urban areas, and populations delineated by 3 age groups: 0–14 years, 15–64 years, and ≥65 years. Furthermore, we considered daily new cases (>10) up to the first reported peak of COVID-19 cases and the corresponding date ranges for all the countries for which such data were available. This range of days varied between 8 and 16 days (Table 1). We use 2 coupled differential equations 
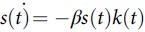
 and 
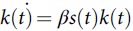
, where *s*(*t*) and *k*(*t*) represent susceptible and infected at time *t*, and *β* is the transmission rate that is assumed to be invariant within the range of days for which the infection numbers in each country were computed. The respective *β* values per 100,000 thousands for the age groups 15–64 years and ≥65+ years considered for various countries are as follows: China: 0.8×1.5  and 1.5, 0.75; Italy: 1.5 and 3.0; Iran: 1.5 and 9.0; South Korea: 2.25 and 4.50; France: 1.50 and 3.0; Spain: 3.0 and 6.0; Germany: 1.5 and 3.0; and the United States: 0.75 and 1.5. The difference between model-predicted numbers and the actual numbers reported within the range were treated as underreported, which includes underdiagnosed cases. We constructed the Meyer wavelets for the reported and adjusted data after adjusting the infected number in the population for underreporting. The Meyer wavelet is a differentiable function, *ψ*(*ω*), which is infinitely differentiable in the domain with a function *u* as follows:
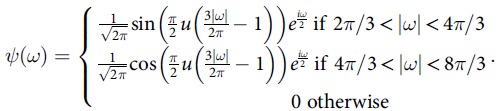



Here, *u*(*x*) = 0 for *x* < 0, *u*(*x*) = *x* for *x* ∈ (0,1), and *u*(*x*) = 1 for *x*1 For further details, please refer to Krantz et al^5^ and Krantz.^9^

As of March 16, 2020, we did not have enough data on COVID-19 transmissibility rates from infected to uninfected persons based on migration of populations to construct countrywide networks. We also had no clear idea of the duration that SARS-CoV-2 virus remains active on nonliving surfaces such as plastics, metals, paper, etc; thus, we did not consider the interaction between humans and nonliving surfaces. Mathematical modeling can be made more complex by adding more parameters, but caution is necessary to ensure that these studies are well designed and that these parameters use readily available, scientifically collected data. Once we obtain more data on the duration of COVID-19 living on nonliving surfaces, we can build more complex models with more parameters.
